# Types and Outcomes of Dietary Interventions in IBS: A Scoping Review

**DOI:** 10.3390/nu18091334

**Published:** 2026-04-23

**Authors:** Bodil Ohlsson, Per M. Hellström, Maria Björklund

**Affiliations:** 1 Department of Clinical Sciences, Malmö, Lund University, 221 00 Lund, Sweden; 2 Department of Internal Medicine, Skåne University Hospital, 205 02 Malmö, Sweden; 3 Department of Medical Sciences, Uppsala University, 751 05 Uppsala, Sweden; per.hellstrom@medsci.uu.se; 4 Faculty of Medicine Library, Lund University, 221 00 Lund, Sweden; maria.bjorklund@med.lu.se

**Keywords:** irritable bowel syndrome, diet, nutrition, outcome, scoping review

## Abstract

Background/Objectives: Irritable bowel syndrome (IBS) is primarily treated via dietary modifications. Several diets have been shown to improve symptoms with similar efficacy. Other aspects of IBS, such as insufficient nutrient intake and being overweight, should also be considered when planning treatment options. The present scoping review aimed to identify various diets investigated in IBS-related clinical trials and to map the measured outcomes. Methods: We performed a systematic search of three databases: PubMed, Embase, and CINAHL Ultimate. Our search was limited to papers published between January 2000 and February 2026, and included human studies published as peer-reviewed original articles in English that described dietary interventions in adult patients (≥18 years) with IBS. Results: The titles and abstracts of 1261 studies were screened; 1147 studies were excluded; and 114 full-text articles were assessed for eligibility. Excluding articles outside the scope of our research resulted in a total of 71 included articles from 57 unique clinical trials. The most common interventions were low fermentable oligo-, di-, and monosaccharides and polyols (FODMAP) (*n* = 43), traditional dietary advice (*n* = 13), and gluten-free diets (*n* = 11). The most common primary outcomes were the effect on IBS symptoms (*n* = 48), efficacy in terms of improving quality of life (*n* = 10), psychological well-being (*n* = 7), nutrient intake (*n* = 7), and adherence/applicability/feasibility to the diet (*n* = 7). Conclusions: In conclusion, the most studied dietary intervention in IBS was low FODMAP, and an effect on GI symptoms was the most common outcome. Considering other conditions associated with IBS, the effects on anthropometric, endocrine, metabolic, and nutritional parameters should also be evaluated.

## 1. Introduction

Irritable bowel syndrome (IBS) is a disorder of gut–brain interaction (DGBI) which is diagnosed based on established symptom criteria [1]. Its etiology and pathophysiology are likely multifactorial, including visceral hypersensitivity, microbial dysbiosis, psychological factors, and low-grade inflammation [2]. In recent years, reports have shown that overweight and obesity are risk factors for IBS [3], and up to 31% of people with obesity have this condition [4], whereas in non-obese people, it has a prevalence of around 9% [5,6]. IBS patients are also more likely to experience metabolic syndrome and prediabetes, exhibiting higher levels of C-peptide and insulin compared with healthy controls [6,7].

Most patients experience worsening symptoms following food intake, leading them to make dietary changes, such as eliminating certain foods [8]. Up to 60% of patients with IBS adhere to some form of dietary restriction [8]. It is well-known that patients with IBS may have inadequate nutritional intake [9]. In comparison to controls, patients with IBS have lower diet quality with decreased intake of protein and micronutrients [10,11]. Vitamin D deficiency is common in IBS patients [12], and vitamin D supplements are the most often studied nutritional supplements in IBS interventions. Systematic reviews and meta-analyses have found that vitamin D supplementation improves quality of life and reduces IBS symptoms [2,13].

The increasing consumption of ultra-processed food (UPF) in recent years is associated with an increased risk of developing IBS [14]. UPF has also been linked to the escalating global burden posed by multiple chronic diseases [15]. It has yet to be determined whether a poor-quality diet leads to bowel symptoms and may cause IBS, or whether the restrictive diets followed by IBS sufferers lead to malnutrition. However, many dietary interventions fail to measure the effects on nutritional status. When measured, the follow-up is normally conducted during a short period with the intervention led by registered dietitians (RDs) or specialists in the field. So far, no study has investigated nutritional balance when restrictive diets are used in regular clinical care or self-administered by the patient. The more complicated the diet, the greater the demands on the patients in terms of their nutritional knowledge, which requires detailed education. Furthermore, the effects on the patient’s metabolism or endocrine profile are seldom assessed.

In addition to their effects on nutritional composition, restrictive diets may also lead to social isolation, a greater risk of psychosocial problems, increased likelihood of disordered eating, and more food-based anxiety [16,17]. Thus, the effects of improved gastrointestinal (GI) symptoms may, contradictorily, have a negative impact on quality of life due to the introduction of challenging food restrictions [18].

Several recommended diets for IBS involve a reduction in various carbohydrates and fats [19,20,21,22], with similar effects on GI symptoms, regardless of food content [21,22,23]. This may partially be due to strong placebo effects in IBS patients [24]. Together, these findings suggest that when various diets have similar effects on GI symptoms in IBS, it should be important to select the diet that is easiest to accomplish, leading to improved quality of life and general well-being, and leading to the greatest improvements in nutritional status and general health. Several systematic reviews and meta-analyses have assessed the dietary effects on IBS symptoms and quality of life (QoL) [25,26,27,28,29,30,31]. A few systematic reviews and meta-analyses highlighted changes in the gut microbiota in their outcome assessments [32,33,34,35]. However, few reviews and meta-analyses have evaluated the effects on endocrine, metabolic, or nutritional profiles during or after dietary intervention. Even if these aspects were addressed, they were not stated as the outcome, which affected their search strategy.

In this scoping review, we therefore first wanted to clarify which diets have been investigated for IBS/DGBI and what characterizes them by mapping and describing their components. However, the main aim was to determine the primary and secondary outcomes of the included studies, to map existing knowledge in the field, and identify research gaps.

## 2. Materials and Methods

This scoping review was performed according to the Preferred Reporting Items for Systematic Reviews and Meta-analyses Extension for Scoping Reviews (PRISMA-ScR) [36] and JBI Manual for Evidence Synthesis for Scoping Reviews [37]. The review protocol was published on FigShare [38]. 

### 2.1. Eligibility Criteria

Inclusion criteria:

Human studies.

Peer-reviewed original articles.

Articles written in English.

Articles that describe dietary interventions in adult patients (≥18 years) with IBS/DGBI.

Exclusion criteria:

Animal studies.

Conference abstracts, reviews, cross-sectional studies, retrospective studies, case reports, and pediatric studies.

Studies on dietary supplements and probiotics.

### 2.2. Information Sources and Search Strategy

A systematic search strategy was developed by the authors, one of whom is an information specialist (MB). Three databases were searched (28 January 2026): PubMed (National Library of Medicine), Embase (Elsevier), and CINAHL Ultimate (EBSCOHost) using a combination of thesaurus terms and relevant keywords. A set of key papers was used to test the sensitivity of the search. The reference lists of the included articles were checked for additional relevant references. If deemed relevant, a citation search was conducted for the included studies. The search was limited to English-language studies published between January 2000 and February 2026. The full search strategy is described in Figure S1 and Text document S1. The PRISMA-ScR checklist was followed when writing the manuscript (Table S1).

### 2.3. Study Selection

Titles and abstracts were screened by two independent investigators using Covidence online software [39]. The full-text articles were then screened for the eligibility criteria by the lead investigator (BO). The exclusion of any article at this stage was confirmed by a second investigator (MB). Any disagreements were resolved by discussion.

### 2.4. Data Extraction

A table was created to describe the study characteristics, such as dietary intervention, location, design, and study population description. All primary and secondary outcomes were listed. Data was extracted by one investigator and exhaustively checked by another to confirm their agreement.

### 2.5. Critical Appraisal

A full risk-of-bias assessment of each included study was not considered necessary for the synthesis; a risk-of-bias assessment is not a mandatory part of scoping reviews, which are expected to assess and understand the extent of knowledge in a field or to identify or map concepts in that field [37]. Critical perspectives on the studies included are provided in the Section 4.

### 2.6. Synthesis

In this scoping review, we aimed to clarify which diets have been investigated for their ability to improve IBS/DGBI symptoms and how they are characterized. We also mapped and described the components of said diets. We provided a synthesis of the primary and secondary outcomes of the included studies to map existing knowledge and identify research gaps.

## 3. Results

A total of 1261 studies were screened after the removal of 257 duplicates. After titles and abstracts were screened, 1147 studies were excluded, and 114 full-text articles were assessed to determine their eligibility. Articles outside the scope of this review were excluded, leading to a final total of 71 included articles from which data were extracted (Figure S1). The included articles met the inclusion criteria, and in all of these papers, the diagnosis of IBS/DGBI was established according to the Rome criteria versions II-IV [1,40,41], with a single exception; in one study, the NICE criteria were used [42,43]. The vast majority of patients had IBS, independent of subtype, but some cohorts also included patients with other types of DGBI, such as functional dyspepsia and less specific functional symptoms [44,45,46].

### 3.1. Dietary Interventions

Fifty-seven unique studies were identified, resulting in a total of 71 published articles. Many of the unique interventions were performed without any randomization or a control group, where the effects before and after the intervention were assessed (*n* = 19). When randomized, most interventions were open, clinical trials (*n* = 19), and some were crossover studies (*n* = 4). Most of the blinded studies were single-blind (*n* = 10), with only one crossover study, whereas some were double-blind (*n* = 9); of these, four were crossover studies (Table 1).

The most common diet examined in the 57 unique interventions was low in fermentable oligo-, di- and monosaccharides, and polyols (FODMAP) (*n* = 43), sporadically modified with a gluten-free diet (GFD) or Mediterranean diet (MD), traditional dietary advice (TDA) according to the National Institute for Health and Care Excellence (NICE) guidelines (*n* = 13), GFD (*n* = 11), MD (*n* = 4), starch- and sucrose-reduced diet (SSRD) (*n* = 3), Tritordeum-based diet (*n* = 3), low-carbohydrate/high-fat diet (LCHF) (*n* = 2), fructose-reduced diet (FRD) (*n* = 2), lactose-reduced diet (*n* = 2), and fiber-rich diet (*n* = 1). Sporadic interventions were performed with specific modifications of carbohydrate content. The effect of low FODMAP was assessed before and after the intervention and during long-term follow-up, and compared with TDA or modified NICE guidelines (*n* = 11), habitual diet (*n* = 4), GFD (*n* = 3), Tritordeum-based diet (*n* = 2), MD (*n* = 2), specific carbohydrate diets (*n* = 2), simplified low FODMAP (*n* = 2), moderately low FODMAP (*n* = 1), high FODMAP (*n* = 1), lactose-free diet (*n* = 1), or SSRD (*n* = 1) (Table 1).

### 3.2. Mapping of Outcomes

The most common primary outcome of the 71 published studies was assessing the effects on IBS symptoms (*n* = 48), with only one study reporting this as a secondary aim. In addition to the effect on GI and IBS symptoms, the impacts on quality of life (primary aim: *n* = 10 and secondary aim: *n* = 1), psychological well-being (primary aim: *n* = 7 and secondary aim: *n* = 1), and extra-intestinal symptoms (primary aim: *n* = 1 and secondary aim: *n* = 2) were estimated (Table 1), and symptoms were self-reported. To assess GI symptoms, the IBS-Severity Scoring System (IBS-SSS) was the most commonly administered questionnaire [112], appearing in 62% of the 71 articles. This questionnaire was also used to assess extra-intestinal symptoms. The Bristol Stool Scale (BSS) was used to measure stool form and consistency in 28% of the studies [113]. Quality of life was most frequently assessed using the IBS Quality of Life (IBS-QOL) scale [114], which was used in 21% of the examined articles. Various questionnaires were used to assess different aspects of psychological well-being, but the Hospital Anxiety and Depression Scale (HADS) was most often used to assess psychological distress (16%) [115].

The dietary effect on nutritional intake was assessed regarding both macro- and micronutrients as a primary outcome in some studies (*n* = 7), as well as a secondary outcome in others (*n* = 3). The circulating levels of albumin and micronutrients were the primary outcome in one single study and the secondary outcome in another (Table 1).

In three studies, the primary aim was to achieve a definitive diagnosis of non-celiac gluten sensitivity (NCGS). The adherence/applicability/feasibility to the diet was examined (primary aim: *n* = 7 and secondary aim: *n* = 1), as well as anthropometric assessments of weight, BMI, body composition, and/or waist and hip circumferences (primary aim: *n* = 4 and secondary aim: *n* = 2). Regarding laboratory analyses of feces, the gut microbiota in the form of diversity and taxonomy levels (primary aim: *n* = 7 and secondary aim: *n* = 1) and short-chain fatty acids (SCFA) (primary aim: *n* = 3 and secondary aim: *n* = 1) were examined. Metabolomics (primary aim: *n* = 4, two from feces and two from blood), and inflammatory biomarkers, e.g., C-reactive protein (CRP), cytokines, and calprotectin (primary aim: *n* = 2 and secondary aim: *n* = 3 and primary aim: *n* = 1 and secondary aim: *n* = 1, respectively) were examined in both blood and feces. Intestinal barrier function (*n* = 3) was assessed based on lactulose and mannitol excretion, circulating and urine markers, as well as mucosal biopsies. Sporadic other outcomes, such as fatigue, sleeping habits, blood pressure, and sugar cravings, were also measured (Table 1).

In blood, the metabolic profile, i.e., HbA1c and lipid levels (primary aim: *n* = 2 and secondary aim: *n* = 1) and endocrine profile, i.e., C-peptide, insulin, copeptin, gastric inhibitory polypeptide (GIP), glucagon-like peptide-1 (GLP-1), glucagon, leptin, luteinizing hormone, plasminogen activator inhibitor-1 (PAI-1), polypeptide YY, and visfatin (*n* = 3) were measured. Autoantibodies against gonadotropin-releasing hormone (GnRH), its precursor, progonadoliberin-2, and its receptor, and tenascin C were measured on one occasion (Table 1).

## 4. Discussion

This scoping review found that most dietary interventions involved a low-FODMAP diet (75%), and the most commonly studied outcome was the effect of this diet on GI and other IBS-related symptoms (68%). The low-FODMAP diet has previously been shown to improve symptoms of IBS [43,57,65,72,79,99,109,116,117] and is best known as a regimen for IBS [118]. In line with this, a few single-blind studies incorporated FODMAP components [79,119,120,121,122,123,124] and similar double-blind studies used provocations with only a few FODMAPs [119,120,121,123,124], resulting in worsening of IBS symptoms. The anthropometric parameters were often assessed in the low FODMAP interventions, but circulating levels of nutrients and inflammatory biomarkers or metabolic and endocrine profiles were seldom stated as outcomes of the studies.

### 4.1. IBS and Metabolic Aspects

The etiology and pathophysiology behind IBS remain undefined [1,2]. For many years, IBS has been associated with metabolic syndrome and prediabetes with high plasma levels of C-peptide, insulin, and triglycerides [6,7,125]. A 12% increased risk of IBS was recorded for each increase in the standard deviation of triglycerides [126]. Overweight respondents report higher pain intensity and pain scores than respondents of a normal weight [127], which could explain the associations described. In a recent systematic review and meta-analysis, as well as in a large-scale database, IBS was associated with an increased risk of metabolic syndrome, insulin resistance, elevated BMI, waist circumference, systolic hypertension, high cholesterol levels, low-density lipoprotein (LDL) cholesterol, and triglycerides, and lower levels of high-density lipoprotein (HDL) cholesterol [128,129]. In a large population-based prospective long-term study, patients with steatotic liver disease had an 11% greater risk of developing IBS. Furthermore, the risk was higher in patients with a certain cardiometabolic risk profile, such as people with metabolic dysfunction-associated steatotic liver disease, suggesting that management of the liver disease may help to prevent IBS [130]. In spite of this, only a few dietary interventions used parameters reflective of metabolic syndrome as outcomes in their studies [12,66,70,91,94,111]. Considering the global epidemic of overweight and subsequent development of metabolic syndrome and diabetes [131,132], this topic is worth studying in future trials. Weight reduction could also be important for reducing IBS symptoms [49] as well as preventing symptom development and IBS/DGBI.

### 4.2. IBS and Nutrition

Dietary habits differ between patients with IBS and healthy controls [10,11,133]. In recent years, researchers have highlighted associations between IBS and the Western diet [134] and UPF [14,108]. Intake of sugar-sweetened beverages has been associated with a proteomic profile connected to type 2 diabetes incidence [135]. Another study discovered a similar metabolic profile in patients with IBS and type 2 diabetes [129]. Thus, poor-quality dietary intake may have a significant influence on causality for both IBS, whose symptoms are markedly affected by food intake, and for the global epidemic of metabolic syndrome [20,23,128]. A low-carbohydrate diet may hold promise for addressing both GI symptoms and cardiometabolic risks in patients with IBS [21,94].

Nutrient insufficiency may cause several symptoms. However, it is still unclear how important nutritional deficiency or insufficiency is for the development of IBS. Selenium deficiency was found to induce IBS in patients whose data were stored in a large UK biobank [136]. Selenium intake increased when sugary foods were replaced with more vegetables, fruit, and dairy products [49], employing SSRD. At the same time, protein, fat, vitamin, and mineral intake increased, and the consumption of fructans and galacto-oligosaccharides (GOS) decreased [49], potentially broadening health benefits and providing a more nutritionally balanced approach with higher nutrient density than the low-FODMAP diet. Along similar lines, epidemiological studies have shown that vitamin D deficiency and subsequent treatment hold great importance with regard to IBS symptoms [2,13]. Thus, there should be a greater focus on improving the nutrient intake of IBS patients, not only to alleviate their symptoms, but also to improve their general health [15].

There are some overlaps between diets, since gluten-free products are recommended as part of a low FODMAP diet [10,19]. Low FODMAP and SSRD diets have also been found to overlap, as the latter diet also leads to reductions in fructan and GOS [49]. The Mediterranean diet shares some common components with low FODMAP and SSRD diets, since all of these diets encourage increased intake of vegetables, legumes, etc. [10,19,21,52,97]. This overlap may explain the difficulties that arise in identifying differences between dietary groups.

### 4.3. IBS and Pathophysiology

Interactions between intestinal endocrine cells and diet in IBS have been described [137]. Nevertheless, the endocrine profile was the outcome in only two of the three unique SSRD studies, which showed lower levels of PAI-1, etc. [96,101]. This is of interest to us, since epidemiological studies support a link between PAI-1 and type 2 diabetes [138]. So far, analyses of the endocrine profile have only been performed in one study with low FODMAP [101]. Despite years of study, no consensus regarding the endocrines importance in IBS has been reached. Considering the significant effect that several metabolic hormones have on gut function and motility, the endocrine response to diet and ensuing effects on the gut deserve further examination regarding how circulating hormone levels contribute to gastrointestinal disorders [101,139].

IBS has been speculated to be associated with low-grade inflammation [140], but circulating levels of inflammatory markers are seldom measured. Some food, e.g., UPF, is suspected to influence disease through inflammatory mediators [15]. Furthermore, chronic, low-grade systemic inflammation, often referred to as “meta-inflammation,” is considered a key pathophysiological basis for insulin resistance and cardiovascular disease [141]. The hypothesis must be raised as to whether the low-grade inflammation in IBS may be due to excess weight [142], high intake of sugar or UPF on a societal level [15], or the IBS pathology per se.

The number of studies on gut microbiota and SCFA is increasing. Dietary effects on the gut microbiota may be one important mediator of the pathophysiology behind IBS. There is growing evidence of a connection between the gut–microbiota–brain, and the gut microbiota has been associated with many diseases [81,85,140].

### 4.4. Strengths and Weaknesses

The strength of the current scoping review is in its systematic selection and synthesis of included studies, which were conducted according to PRISMA-ScR [36]. One of the limitations of our research is that we have not calculated the effects of the studies. However, this was not the primary scope of this study, since we wanted to map the actual diets examined and the different outcomes of the included studies. Another limitation is that only studies in English were included, which may have introduced bias, leading to distortion of the data. No risk-of-bias assessment analysis was performed, since this is not a mandatory part of scoping reviews [37]. In general, the intention of a systematic review is to present information about clinical decision-making, whereas scoping reviews are more appropriate for assessing and understanding the extent of the knowledge in a field or identifying and reporting characteristics or concepts in that field [37]. Thus, even if some articles are not published, it may not lead to serious consequences. There is always a risk of selection bias, even if a systematic search strategy is used. To minimize this, the inclusion and exclusion criteria were well-defined before the study started, and the literature screening was performed by two independent investigators. Further, when only the interventions used and the intended outcomes are studied, the quality of the inclusion mode and the article are of lower importance than when effect sizes are assessed.

### 4.5. Future Perspectives

When different diets have equal effects on GI symptoms and quality of life [23,143], it is important to consider other aspects of food intake and recommended dietary strategies. More studies are warranted to investigate adherence and the feasibility of the diets in non-hospital settings and without access to a skilled dietitian. The great improvement in micronutrient intake following SSRD compared with low FODMAP should be considered in future studies and discussed further in recommendation guidelines [49]. To assess quality of life along with GI symptoms, it is necessary to understand the more general relief of symptom burden for patients [59]. Although the weight/BMI or the metabolic syndrome parameters were measured in several studies, the absence of these factors in the outcome means that the studies are not powered or designed for the correct analysis of these parameters. Furthermore, the data were not fully analyzed to explore associations and effects.

## 5. Conclusions

The most studied dietary intervention in the IBS context is low FODMAP, and effects on GI symptoms were the most common outcomes. There is a need for more studies that investigate the adherence to and feasibility of such diets outside the healthcare system, and the effects on nutrient intake when patients do not have access to professional healthcare providers. The effects of diets on anthropometric, endocrine, and metabolic parameters should also be studied, as this will offer a more holistic view of DGBI, a disorder that is not solely restricted to GI symptoms. More aspects must be considered when preparing treatment guidelines, in addition to direct effects on evaluable GI symptoms.

## Figures and Tables

**Table 1:** Studies included in the scoping review.

Authors	Title	Country	Study Design	Diet	N	Aim of Study and Outcomes
Algera et al., 2022 [47]	Low FODMAP diet reduces gastrointestinal symptoms in irritable bowel syndrome and clinical response could be predicted by symptom severity: A randomized crossover trial	Sweden	Randomized, double-blind crossover intervention	Low FODMAP vs. moderate FODMAP	29	Primary aim: identify dietary effects on GI symptoms Secondary aims: identify effects on other somatic symptoms, psychological distress, and determine predictors of clinical response
Aller et al., 2004 [48]	Effects of a high-fiber diet on symptoms of irritable bowel syndrome: a randomized clinical trial	Spain	Randomized, single-blind intervention	Low or high fiber content	46	Examine the effect of dietary fiber on IBS symptoms
Al-Shiblawi et al., 2025 [49]	A starch- and sucrose-reduced diet leads to a more favorable nutrient profile than low FODMAP in patients with irritable bowel syndrome. A randomized clinical trial	Sweden	Randomized, non-inferiority intervention	Low FODMAP vs. SSRD	155	Primary aim: assess nutritional intake before and after intervention and at 6-month follow-up Secondary aims: associations between changes in nutrient intake, weight, and symptom patterns
Austin et al., 2009 [50]	A very low-carbohydrate diet improves symptoms and quality of life in diarrhea-predominant irritable bowel syndrome	USA	Non-randomized intervention	Very-low-carbohydrate diet (VLCD)	17	Adequate relief of IBS-D symptoms during the VLCD phase
Aziz et al., 2016 [51]	Efficacy of a gluten-free diet in subjects with irritable bowel syndrome-diarrhea unaware of their HLA-DQ2/8 genotype	United Kingdom	Non-randomized intervention	Gluten-free diet (GFD)	41	Primary aim: clinical response to GFD Secondary aim: maintaining GFD was safe, sustainable, and beneficial
Bamidele et al., 2025 [52]	The Mediterranean diet for irritable bowel syndrome: A randomized clinical trial	United Kingdom	Randomized non-inferiority intervention.	Mediterranean diet (MD) vs. TDA	139	Determine if the MD is non-inferior to TDA in managing IBS symptoms
Barone et al., 2020 [53]	Evaluation of non-celiac gluten sensitivity in patients with previous diagnosis of irritable bowel syndrome: A randomized double-blind placebo-controlled crossover trial	Italy	Randomized, double-blind, placebo-controlled, crossover intervention	Low FODMAP-GFD; those with improvement were exposed to gluten vs. placebo	30	Achieve a definitive diagnosis of non-celiac gluten sensitivity (NCGS) in IBS patients
Bellini et al., 2020 [54]	A low-FODMAP diet for irritable bowel syndrome: some answers to the doubts from a long-term follow-up	Italy	Long-term follow-up of the low FODMAP diet	Low FODMAP	73	Primary aim: determine LFD’s short- and long-term effects on abdominal symptoms, QoL, anxious–depressive symptoms, quality of sleep, degree of relief, and degree of patient satisfaction in IBS patients Secondary aim: adherence to the LFD, their acceptability, food-related QoL life and ability to reliably identify FODMAP foods
Berg et al., 2013 [55]	Effect of fructose-reduced diet in patients with irritable bowel syndrome, and its correlation to a standard fructose breath test	Norway	Randomized intervention	IBS diet with or without additional FRD	182	Primary aim: precision of the fructose breath test (FBT) with symptom score reduction on FRD Secondary aim: clinical utility of the FBT in comparison with an empirical diagnosis of FM
Bjørkevoll et al., 2025 [56]	A 12-week strict low FODMAP diet reduces the severity levels of fatigue, depression, anxiety, and inattention in patients with irritable bowel syndrome	Norway	Non-randomized intervention	Low FODMAP	36	Changes in symptoms of fatigue, depression, anxiety, and attention-related performance
Böhn et al., 2015 [57]	Diet low in FODMAPs reduces symptoms of irritable bowel syndrome as well as traditional dietary advice: a randomized controlled trial	Sweden	Randomized, single-blind intervention	Low FODMAP vs. TDA	75	Compare the dietary effect on IBS symptoms
Caponio et al., 2022 [58]	Evaluation of the effects of the Tritordeum-based diet compared to the low-FODMAPs diet on the fecal metabolome of IBS-D patients: A preliminary investigation	Italy	Randomized intervention	Low FODMAP vs. Tritordeum-based diet	38	Effect of 12 weeks of either diet on the fecal metabolomic profile of patients with IBS-D
Chavan et al., 2025 [59]	Comparative study on the impact of different dietary interventions on symptoms and quality of life in patients with irritable bowel syndrome	India	Randomized intervention	Low FODMAP, GFD, or TDA	150	The impact of three distinct dietary interventions on symptom severity and QoL
Cingolani et al., 2020 [60]	Feasibility of low fermentable oligosaccharide, disaccharide, monosaccharide, and polyol diet and its effects on quality of life in an Italian cohort	Italy	Non-randomized intervention	Low FODMAP	60	Determine which IBS patients could benefit more from FODMAP reduction and examine the feasibility and applicability of such a dietary plan.
Clevers et al., 2020 [61] Same as Böhn et al., 2015 [57]	Adherence to diet low in fermentable carbohydrates and traditional diet for irritable bowel syndrome	Sweden	Randomized single-blind intervention, post hoc	Low FODAMP vs. TDA	66	Adherence to the low FODMAP and traditional IBS diet and related dietary changes to symptom improvement in IBS patients
de Roest et al., 2013 [62]	The low FODMAP diet improves gastrointestinal symptoms in patients with irritable bowel syndrome: a prospective study	New Zealand	Non-randomized intervention	Low FODMAP	90	Determine prospectively whether a low-FODMAP diet leads to improved symptoms
Elli et al., 2016 [44]	Evidence for the presence of non-celiac gluten sensitivity in patients with functional gastrointestinal symptoms: Results from a multicenter randomized double-blind placebo-controlled gluten challenge	Italy	Randomized, multicenter, double-blind, crossover intervention	GFD vs. gluten-containing diet	140	Identify patients with non-celiac gluten sensitivity (NCGS) from those reporting an improvement of GI symptoms after GFD
Eswaran et al., 2016 [63]	A randomized controlled trial comparing the low FODMAP diet vs. modified NICE guidelines in US adults with IBS-D	USA	Randomized, single-blind intervention	Low FODMAP vs. modified NICE guidelines	92	Compare the efficacy of diets overall and for individual symptoms in IBS-D patients
Eswaran et al., 2020 [64] Same as Eswaran et al. 2016 [63]	The Impact of a 4-week low-FODMAP and mNICE diet on nutrient intake in a sample of US adults with irritable bowel syndrome with diarrhea	USA	Post hoc analysis of a randomized, single-blind intervention	Low FODMAP vs. modified NICE guidelines	78	Determine changes in the mean daily nutrient content before and after LFD and identify nutritional inadequacies, comparing them with NICE guidelines
Eswaran et al., 2017 [65] Same as Eswaran et al. 2016 [63]	A diet low in fermentable oligo-, di-, and Monosaccharides and polyols improves quality of life and reduces activity impairment in patients with irritable bowel syndrome and diarrhea	USA	Randomized, single-blind intervention	Low FODMAP vs. modified NICE guidelines	92	Whether a low-FODMAP diet would improve disease-specific QOL, psychological distress, work productivity, and sleep to a greater degree than standard dietary recommendations
Frieling et al., 2019 [66]	Tolerability of FODMAP—reduced diet in irritable bowel syndrome—efficacy, adherence, and body weight course	Germany	Non-randomized intervention	Low FODMAP	93	Effects on symptoms, weight, and adherence of the low FODMAP diet
Gayoso et al., 2023 [67]	The effect of starch- and sucrose-reduced diet accompanied by nutritional and culinary recommendations on the symptoms of irritable bowel syndrome patients with diarrhea	Spain	Non-randomized intervention	SSRD	34	Effect of SSRD on symptoms, QoL, and nutritional habits of patients with IBS-D
Goyal et al., 2021 [68]	Low fermentable oligosaccharide, disaccharide, monosaccharide, and polyol diet in patients with diarrhea-predominant irritable bowel syndrome: A prospective, randomized trial	India	Randomized, single-blind intervention	Low FODMAP vs. TDA	101	Efficacy and acceptability of short-term strict LFD and long-term FODMAP diet in patients with IBS-D
Goyal et al., 2025 [45]	Prevalence and predictors of nonceliac wheat sensitivity in refractory irritable bowel syndrome and functional dyspepsia: results from a randomized double-blind placebo-controlled study	India	Randomized double-blind, placebo-controlled with crossover	GFD vs. gluten-containing diet	177	Determine the prevalence, clinical predictors, and impact of GFD in IBS
Gravina et al., 2020 [69]	Adherence and effects derived from FODMAP diet on irritable bowel syndrome: A real-life evaluation of a large follow-up observation	Italy	Non-randomized intervention	Low FODMAP	120	Adherence to a 6-week diet and its long-term effects on IBS symptoms
Guerreiro et al., 2020 [70]	Effectiveness of two dietary approaches on the quality of life and gastrointestinal symptoms of individuals with irritable bowel syndrome	Portugal	Non-randomized intervention	Low FODMAP vs. habitual diet	70	Symptoms, QoL, and nutritional status before and after intervention
Hajiani et al., 2019 [71]	Comparison between gluten-free regime and regime with gluten in symptoms of patients with irritable bowel syndrome (IBS)	Iran	Randomized, double-blind intervention	GFD vs. habitual diet	140	Effect of gluten on IBS symptoms
Halmos et al., 2014 [72]	A diet low in FODMAPs reduces symptoms of irritable bowel syndrome	Australia	Randomized, single-blind, crossover intervention	Low FODMAP vs. habitual diet	38	To fill the major gaps in evidence for the efficacy of the low-FODMAP diet
Harvie et al., 2017 [73]	Long-term irritable bowel syndrome symptom control with reintroduction of selected FODMAPs	New Zealand	Randomized, crossover intervention	Low FODMAP vs. habitual diet	50	Long-term effect of dietary education on FODMAP intake, nutritional adequacy, symptom severity, QoL, and reduction in the GI microbiome
Ibrahim & Stribling 2019 [46]	A 5Ad dietary protocol for functional bowel disorders	United Kingdom	Non-randomized intervention	5Ad dietary protocol	22	Efficacy in reducing GI symptoms
Jensen et al., 2025 [74]	Modification of the low FODMAP diet is feasible in the treatment of irritable bowel syndrome: A randomized crossover study	Denmark	Randomized crossover intervention	Low FODMAP vs. modified low FODMAP	9	Examine if all carbohydrate groups eliminated in the low-FODMAP diet are equally important for relieving GI symptoms.
Kasti et al., 2025 [75]	Clinical trial: A Mediterranean low-FODMAP diet alleviates symptoms of non-constipation IBS-randomized controlled study and volatomics analysis	Greece	Randomized intervention	MED-LFD vs. TDA	108	Assess effectiveness for managing IBS symptoms and improving QoL and highlight potential pathophysiological insights by evaluating stool SCFAs/branched-chain fatty acids (BCFAs)
Kortlever et al., 2019 [76]	Low-FODMAP diet is associated with improved quality of life in IBS patients—A prospective observational study	Australia and New Zealand	Non-randomized intervention	Low FODMAP	111	Assess QoL, depression, anxiety, vitality, and happiness in patients with IBS
Krieger-Grübel et al., 2020 [77]	Treatment efficacy of a low FODMAP diet compared to a low lactose diet in IBS patients: A randomized, cross-over designed study	Switzerland	Randomized, crossover intervention	Low FODMAP vs. low lactose diet (LLD)	29	Examine if the demanding LFD is more effective than elimination of lactose alone
Linsalata et al., 2021 [78]	The relationship between low serum vitamin D levels and altered intestinal barrier function in patients with IBS diarrhea undergoing a long-term low-FODMAP diet: Novel observations from a clinical trial	Italy	Non-randomized intervention	Low FODMAP	36	GI symptoms and urinary and circulating markers of intestinal barrier function and integrity in IBS-D patients with vitamin D deficiency (L-VD) compared to those with normal VD levels (N-VD)
McIntosh et al., 2017 [79]	FODMAPs alter symptoms and the metabolome of patients with IBS: a randomized controlled trial	Canada	Randomized, single-blind intervention	Low vs. high FODMAP	37	Impact of FODMAPs on the metabolome gut microbiota composition and GI symptoms
Moritz et al., 2013 [80]	Effect of a fructose and lactose elimination diet in patients with irritable bowel syndrome: A randomized double-blind placebo-controlled study	Austria	Randomized double-blind placebo-controlled intervention	Fructose-free diet vs. lactose-free diet	320	Whether lactose or fructose elimination diets can improve symptoms in patients with IBS
Naseri et al., 2021 [81]	Influence of low FODMAP-gluten free diet on gut microbiota alterations and symptom severity in Iranian patients with irritable bowel syndrome	Iran	Non-randomized intervention	Low FODMAP-GFD	42	Effects of low FODMAP-GFD on clinical symptoms, intestinal microbiota diversity, and fecal calprotectin (FC) level in patients with IBS
Nilholm et al., 2019 [82]	Irregular dietary habits with a high intake of cereals and sweets are associated with more severe gastrointestinal symptoms in IBS patients	Sweden	Randomized intervention	SSRD vs. habitual diet	105	Effects of SSRD on GI symptoms
Nilholm et al., 2019 [83] Same as Nilholm et al., 2019 [82]	A dietary intervention with reduction of starch and sucrose leads to reduced gastrointestinal and extra-intestinal symptoms in IBS patients	Sweden	Randomized intervention	SSRD vs. habitual diet	105	Effect of SSRD on overall GI symptoms, psychological well-being, and extra-intestinal symptoms
Nilholm et al., 2021 [84] Same Nilholm et al., 2019 [82]	Assessment of a 4-week starch- and sucrose-reduced diet and its effects on gastrointestinal symptoms and inflammatory parameters among patients with irritable bowel syndrome	Sweden	Randomized intervention	SSRD vs. habitual diet	105	Primary aim: effect of SSRD on GI symptoms. Secondary aims: quantify and correlate significant changes in nutrient intakes, CRP and cytokine levels, and GI symptom
Nilholm et al., 2022 [85] Same as Nilholm et al., 2019 [82]	A starch- and sucrose-reduced dietary intervention in irritable bowel syndrome patients produced a shift in gut microbiota composition along with changes in phylum, genus, and amplicon sequence variant abundances, without affecting the micro-RNA levels	Sweden	Randomized intervention	SSRD vs. habitual diet	105	Effects of SSRD on gut microbiota and miRNA composition in IBS, in relation to nutrient intake and GI symptoms
Nybacka et al., 2021 [86]	Changes in serum and urinary metabolomic profile after a dietary intervention in patients with irritable bowel syndrome	Sweden	Randomized intervention	Low FODMAP vs. TDA	53	Identify metabolite patterns related to dietary intake, and identify metabolites driving the separation between responders and non-responders to treatment
Nybacka et al., 2024 [20]	A low FODMAP diet plus traditional dietary advice versus a low-carbohydrate diet versus pharmacological treatment in irritable bowel syndrome (CARIBS): a single-center, single-blind, randomized controlled trial	Sweden	Randomized, single-blind intervention	Low FODMAP plus TDA vs. low-carbohydratediet vs. pharmacologic treatment	294	Effects on IBS symptoms
O’Connor et al., 2025 [87]	Impact of HADS anxiety and depression scores on the efficacy of dietary interventions for irritable bowel syndrome	Ireland	Non-randomized intervention	First TDA and then low FODMAP	448	Primary aim: symptomatic response Secondary aim: symptom response in relation to anxiety and depression scores
Paduano et al., 2019 [88]	Effect of three diets (low-FODMAP, gluten-free and balanced) on irritable bowel syndrome symptoms and health-related quality of life	Italy	Non-randomized intervention	Low FODMAP, GFD, and Mediterranean diet	42	Efficacy of diets in improving IBS patients’ health-related QoL, increasing stool solidity, reducing abdominal bloating and pain, and examining acceptance, adherence, and feasibility
Patcharatrakul et al., 2019 [89]	Effect of structural individual low-FODMAP dietary advice vs. brief advice on a commonly recommended diet on IBS symptoms and intestinal gas production	Thailand	Randomized intervention	Low FODMAP	62	Efficacy of two low-FODMAP dietary advice methods—commonly recommended and structured individual—on IBS symptoms and postprandial hydrogen (H2) and methane (CH4) gas production
Rej et al., 2022 [90]	Efficacy and acceptability of dietary therapies in non-constipated irritable bowel syndrome: A randomized trial of traditional dietary advice, the low FODMAP diet, and the gluten-free diet	United Kingdom	Randomized intervention	Low FODMAP, TDA, or GFD	101	Primary aim: efficacy and acceptability of LFD and GFD against TDA. Secondary aim: acceptability, nutritional, and stool microbial changes associated with these diets
Riezzo et al., 2023 [91]	A Tritordeum-based diet for female patients with diarrhea-predominant irritable bowel syndrome: Effects on abdominal bloating and psychological symptoms	Italy	Non-randomized intervention	Tritordeum-based diet (TBD)	40	Determine the effects the TBD has on symptom relief, together with possible modifications in the anthropometric, nutritional, and psychological profiles in female IBS-D patients who do not respond to standard drug therapies
Roth et al., 2022 [92] Same as Nilholm et al., 2019 [82]	A starch- and sucrose-reduced diet in irritable bowel syndrome leads to lower circulating levels of PAI-1 and visfatin: A randomized controlled study	Sweden	Randomized intervention	SSRD vs. habitual diet	105	Primary aim: determine the blood levels of hormones before and after SSRD Secondary aims: compare levels of hormones and AXIN1 with healthy volunteers and correlate differences in hormonal levels with changes in GI symptoms, psychological well-being, sweet craving, nutritional intake, and weight
Roth et al., 2024 [21]	A starch- and sucrose-reduced diet has similar efficiency as low FODMAP in IBS—A randomized non-inferiority study	Sweden	Randomized, non-inferior intervention	Low FODMAP vs. SSRD	155	Primary aim: determine the effect of either diet on GI symptoms Secondary aims: examine the responder rates after 6 months and with score reductions of ≥100 or ≥50% in IBS-SSS from baseline; the effects on extra-intestinal symptoms; saturation; sugar craving; anthropometric parameters; and blood pressure
Roth and Ohlsson 2025 [93] Merge of Nilholm et al. 2019 [82] and Roth et al. 2024 [21]	Dietary modifications in IBS lead to reduced symptoms, weight, and lipid levels: Two randomized clinical trials	Sweden	Two randomized interventions were merged together	SSRD vs. low FODMAP or habitual diet	260	Primary aim: determine the symptoms, weight, BMI, and lipid levels before and after a dietary intervention and evaluate the associations between nutrient intake and these parameters Secondary aim: compare the effects of either diet regarding nutrient intake, symptoms, weight/BMI, and lipids
Roth et al., 2026 [94] Same as Roth et al., 2024 [21]	Improved lipid and glycemic profile and vitamin D levels after a dietary intervention with SSRD and low FODMAP in IBS patients: A randomized controlled trial	Sweden	Randomized, non-inferior intervention	Low FODMAP vs. SSRD	155	Primary aim: assess the levels of albumin, CRP, HbA1c, lipids, and micronutrients before and after intervention Secondary aim: correlate changes with changes in dietary intake, weight, symptoms, and physical activity
Russo et al., 2022 [95]	A comparison of the low-FODMAPs diet and a Tritordeum-based diet on the gastrointestinal symptom profile of patients suffering from irritable bowel syndrome-diarrhea variant (IBS-D): A randomized controlled trial	Italy	Randomized, single-blind intervention	Low FODMAP vs. Tritordeum-based foods (TBD)	104	Compare the beneficial effects of either diet on improving the symptom profile of IBS-D patients
Saidi et al., 2020 [96]. Same as Nilholm et al. 2019 [82]	A carbohydrate-restricted diet for patients with irritable bowel syndrome lowers serum C-peptide, insulin, and leptin without any correlation with symptom reduction	Sweden	Randomized intervention	SSRD vs. habitual diet	105	Primary aim: hormonal serum concentrations before and after intervention Secondary aims: correlate hormonal changes, carbohydrate intake, and GI symptoms, and measure autoantibodies
Singh et al., 2025 [97]	Efficacy of Mediterranean diet vs. low-FODMAP diet in patients with nonconstipated irritable bowel syndrome: A pilot randomized controlled trial	USA	Randomized intervention	Low FODMAP vs. MD	26	Clinical efficacy of either diet in treating the symptoms of patients with IBS
Singh et al., 2025 [98]	Is a simplified, less restrictive low FODMAP diet possible? Results from a double-blind, pilot randomized controlled trial	USA	Randomized, double-blind intervention	Low FODMAP vs. simple low FODMAP	35	Primary aim: proportion of subjects meeting a >30% reduction in weekly average of daily abdominal pain intensity Secondary aims: proportion of subjects with a reduction regarding other parameters in symptom scores
Staudacher et al., 2011 [43]	Comparison of symptom response following advice for a diet low in fermentable carbohydrates (FODMAPs) versus standard dietary advice in patients with irritable bowel syndrome	United Kingdom	Non-randomized intervention	Low FODMAP and TDA	82	Clinical effectiveness of the low-FODMAP diet with TDA for dietary therapy in IBS
Staudacher et al., 2012 [99]	Fermentable carbohydrate restriction reduces luminal bifidobacteria and gastrointestinal symptoms in patients with irritable bowel syndrome	United Kingdom	Randomized intervention	Low FODMAP vs. habitual diet	41	Effects of FODMAP restriction on the luminal microbiota, SCFA, and GI symptoms
Stenlund et al., 2021 [100] Same as Nilholm et al. 2019 [82]	Metabolic profiling of plasma in patients with irritable bowel syndrome after a 4-week starch- and sucrose-reduced diet	Sweden	Randomized intervention	SSRD vs. habitual diet	105	Primary aim: metabolic profile in relation to SSRD dietary advice Secondary aims: the relationship between metabolic profile, daily nutrient intake, and symptoms
Szekely et al. 2025 [101] Same as Roth et al., 2024 [21]	Leptin and PAI-1 levels are decreased after a dietary intervention in patients with irritable bowel syndrome	Sweden	Randomized intervention	Low FODMAP vs. SSRD	155	Primary aim: confirm whether concentrations of copeptin, leptin, PAI-1, C-peptide, and insulin would change before and after intervention Secondary aim: determine whether hormonal changes could provide insights into underlying mechanisms for symptom alleviation
Tack et al., 2026 [102]	A study on the utility of inflammatory biomarkers in primary care irritable bowel syndrome: a sub analysis of the DOMINO randomized trial	Belgium	Randomized intervention	Low FODMAP vs. Otilonium bromide	472	Primary aim: explore levels of several inflammatory markers in recently diagnosed IBS patients Secondary aims: assess the potential utility of those biomarkers in the diagnosis and prediction of treatment response in this cohort
Tuck et al., 2022 [103]	Changes in signaling from fecal neuroactive metabolites following dietary modulation of IBS pain	Canada	Randomized crossover intervention	Low FODMAP vs. HFD	28	Test the hypothesis that pain relief following reduced consumption of fermentable carbohydrates is the result of changes in luminal neuroactive metabolites
Valeur et al., 2016 [104]	Fecal fermentation in irritable bowel syndrome: Influence of dietary restriction of fermentable oligosaccharides, disaccharides, monosaccharides and polyols	Norway	Non-randomized intervention	Low FODMAP	63	Determine the effect of FODMAP restriction on fecal fermentation. Specifically, the ability of feces to produce SCFAs in vitro was assessed before and after starting a low-FODMAP diet
Vazquez-Roque et al., 2013 [105]	A controlled trial of gluten-free diet in patients with irritable bowel syndrome-diarrhea: effects on bowel frequency and intestinal function	USA	Randomized, double-blind intervention	GFD vs. gluten-containing diet	45	Primary aim: effects of a gluten-containing diet compared to GFD in HLA-DQ2/8-positive and -negative patients with IBS-D regarding bowel function, gut transit, and barrier functions Secondary aims: mucosal morphology and immune activation in response to diets, and the proliferative and cytokine responses of peripheral blood mononuclear cells to gluten and rice antigens.
Vincenzi et al., 2017 [106]	Effects of a Low FODMAP diet and specific carbohydrate diet on symptoms and nutritional adequacy of patients with irritable bowel syndrome: Preliminary results of a single-blinded randomized trial	Italy	Randomized, single-blind intervention	Low FODMAP vs. specific carbohydrate diet	73	Primary aim: assess the efficacy of a low-FODMAP diet on IBS symptoms as compared to a specific carbohydrate diet Secondary aims: evaluate the nutritional adequacy of the two diets and changes in vitamin D and folic acid serum concentration
Wilson et al., 2022 [107]	Fecal and urine metabolites, but not gut microbiota, may predict response to low FODMAP diet in irritable bowel syndrome	United Kingdom	Randomized, single-blind intervention	Low FODMAP vs. habitual diet	69	Assess the differences in fecal microbiota and fecal and urine metabolites between responders and non-responders to the LFD.
Wu et al. 2016 [108] Same as Vasquez-Rogue 2013 et al. [105]	Gluten-induced symptoms in diarrhea-predominant irritable bowel syndrome are associated with increased myosin light chain kinase activity and claudin-15 expression	USA	Randomized, double-blind intervention	GFD vs. Gluten-containing diet	55	To examine three claudin proteins known to be important to intestinal physiology and barrier function
Zahedi et al., 2018 [109]	Low fermentable oligo-di-mono-saccharides and polyols diet versus general dietary advice in patients with diarrhea-predominant irritable bowel syndrome: A randomized controlled trial	Iran	Randomized, single-blind intervention	Low FODMAP vs. TDA	110	Assess the effect of either diet on QoL and clinical symptoms in patients with IBS-D.
Zanwar et al., 2016 [110]	Symptomatic improvement with gluten restriction in irritable bowel syndrome: a prospective, randomized, double blinded placebo-controlled trial	India	Randomized, double-blind intervention	GFD vs. Gluten-containing diet	180	Examine the effect of gluten on GI symptoms
Zhang et al., 2021 [111]	Low fermentable oligosaccharides, disaccharides, monosaccharides, and polyols diet compared with traditional dietary advice for diarrhea-predominant irritable bowel syndrome: a parallel-group, randomized controlled trial with analysis of clinical and microbiological factors associated with patient outcomes	China	Randomized intervention	Low FODMAP vs. TDA	108	Primary aim: assess the efficacy of either diet Secondary aims: identify the clinical, metabolic, and microbiological factors that predict response to dietary interventions in patients with IBS-D

FBT = fructose breath test; FRD = fructose-reduced diet; GFD = gluten-free diet; GI = gastrointestinal; HFD = high-fermentable-carbohydrate diet; IBS = irritable bowel syndrome; low FODMAP = low fermentable oligo-, di-, and monosaccharides and polyols content; QoL = quality of life; SCFA = short-chain fatty acids; SSRD = starch- and sucrose-reduced diet; TDA = traditional dietary advice.

## Data Availability

No new data were created or analyzed in this study, Data Sharing is not applicable to this article.

## Supplementary Materials

The following supporting information can be downloaded at https://www.mdpi.com/article/10.3390/nu18091334/s1, Figure S1: PRISMA flow chart; Table S1: PRISMA-ScR checklist; Text document S1 Search strategy.

## Abbreviations

The following abbreviations are used in this manuscript:

FBT	Fructose breath test.
FRD	Fructose-reduced diet.
GFD	Gluten-free diet.
GI	Gastrointestinal.
GOS	Galacto-oligosaccharides.
HFD	High fermentable carbohydrate diet.
IBS	Irritable bowel syndrome.
LCHF	Low-carbohydrate high-fat diet.
Low FODMAP	Fermentable oligo-, di- and monosaccharides and polyols.
MD	Mediterranean diet.
NICE	National Institute for Health and Care Excellence.
QoL	Quality of life.
SCFA	Short-chain fatty acids.
SSRD	Starch- and sucrose-reduced diet.
TDA	Traditional dietary advice.
UPF	Ultra-processed food.
