# Supplementary material for: Types and Outcomes of Dietary Interventions in IBS: A Scoping Review
Source: Nutrients. 2026 Apr 23;18(9):1334. doi: 10.3390/nu18091334 (PMC13164966; doi:10.3390/nu18091334)
Supplement: Supplementary file 1 [file nutrients-18-01334-s001.zip › Text document S1 Search strategy.pdf]

Search report

Types and outcomes of dietary interventions in IBS: A scoping review

PubMed (National Library of Medicine, 1946-present)

Date of search: January 28, 2026
[Population/problem]
#1 ((("irritable bowel syndrome"[MeSH Terms]) OR ("irritable bowel syndrome/diet therapy"[MeSH Terms])) OR ("brain gut axis"[MeSH Terms])) OR (Irritable Bowel Syndrome[Title/Abstract] OR IBS[Title/Abstract] OR DGBI[Title/Abstract] OR "disorder of gut-brain interaction"[Title/Abstract] OR Functional Gastrointestinal Disorder*[Title/Abstract] OR FGID[Title/Abstract] OR (Brain-gut[Title/Abstract] AND (interaction[Title/Abstract] OR disorder*[Title/Abstract] OR dysfunct*[Title/Abstract])))
27241 records

[Intervention/exposure]
#2 (((((((((("diet"[MeSH Terms]) OR ("feeding behavior"[MeSH Terms])) OR ("diet therapy"[MeSH Terms])) OR ("diet, carbohydrate restricted"[MeSH Terms])) OR ("diet, mediterranean"[MeSH Terms])) OR ("diet, gluten free"[MeSH Terms])) OR ("fodmap diet"[MeSH Terms])) OR (nutritional status[MeSH Terms])) OR ("nutrition therapy"[MeSH Terms])) OR ("malnutrition"[MeSH Terms])) OR (Diet*[Title/Abstract] OR eating[Title/Abstract] OR meal*[Title/Abstract] OR nutrition[Title/Abstract] OR nutritient*[Title/Abstract] OR malnutrit*[Title/Abstract] OR food*[Title/Abstract] OR FODMAP*[Title/Abstract] OR low-FODMAP[Title/Abstract] OR (Fermentable[Title/Abstract] AND (saccharide[Title/Abstract] OR oligosaccharide[Title/Abstract] OR disaccharide[Title/Abstract] OR monosaccharide[Title/Abstract])) OR LFD[Title/Abstract] OR carbohydrate*[Title/Abstract] OR mediterranean[Title/Abstract] OR starch[Title/Abstract] OR sucrose[Title/Abstract] OR ultra-processed[Title/Abstract] OR gluten*[Title/Abstract] OR lactose*[Title/Abstract] OR dairy*[Title/Abstract] OR fiber*[Title/Abstract])
2342300 records

#3 #1 AND #2
5850 records

[Study design filter]
#4 (((((((("clinical trial"[Publication Type]) OR ("controlled clinical trial"[Publication Type])) OR ("randomized controlled trial"[Publication Type])) OR ("clinical trial protocols as topic"[MeSH Terms])) OR ("controlled clinical trials as topic"[MeSH Terms])) OR ("randomized controlled trials as topic"[MeSH Terms]))) OR ("case control studies"[MeSH Terms])) OR ("cohort studies"[MeSH Terms]) OR (Clinical trial OR trial OR “controlled clinical trial” OR randomized OR randomised OR RCT OR case-control OR “case control” OR cohort*)
6065503 records

#5 (((((((("review"[Publication Type]) OR ("systematic review"[Publication Type])) OR ("scoping review"[Publication Type])) OR ("meta analysis"[Publication Type])) OR ("case reports"[Publication Type])) OR ("editorial"[Publication Type])) OR ("review literature as topic"[MeSH Terms])) OR ("systematic reviews as topic"[MeSH Terms])) OR ("scoping

review as topic"[MeSH Terms])
7021270 records
[Combination search]

#6 #3 AND #4
1947 records
#7 #6 NOT #5
1376 records
#8 #7 NOT animal*
1281 records
#9 #8 NOT child*
1107 records

Filter Publication date 2000-present
1017 records
Filter English
979 records

Embase (Elsevier via Embase.com)

Date of search: January 28, 2026

[Population/Problem]
#1 'irritable colon'/exp OR 'brain-gut axis'/exp OR 'irritable bowel syndrome':ab,ti OR ibs:ab,ti OR dgbi:ab,ti OR 'disorder of gut-brain interaction':ab,ti OR 'functional gastrointestinal disorder*':ab,ti OR fgid:ab,ti OR ('brain gut':ab,ti AND (interaction:ab,ti OR disorder*:ab,ti OR dysfunct*:ab,ti))
57515 records
[Intervention/Exposure]
#2 'diet'/exp OR 'feeding behavior'/exp OR 'diet therapy'/exp OR 'low carbohydrate diet'/exp OR 'mediterranean diet'/exp OR 'gluten free diet'/exp OR 'fodmap diet'/exp OR 'nutritional status'/exp OR 'malnutrition'/exp OR diet*:ab,ti OR eating:ab,ti OR meal*:ab,ti OR nutrition:ab,ti OR nutritient*:ab,ti OR malnutrit*:ab,ti OR food*:ab,ti OR fodmap*:ab,ti OR 'low fodmap':ab,ti OR (fermentable:ab,ti AND (saccharide:ab,ti OR oligosaccharide:ab,ti OR disaccharide:ab,ti OR monosaccharide:ab,ti)) OR lfd:ab,ti OR carbohydrate*:ab,ti OR mediterranean:ab,ti OR starch:ab,ti OR sucrose:ab,ti OR 'ultra processed':ab,ti OR gluten*:ab,ti OR lactose*:ab,ti OR dairy*:ab,ti OR fiber*:ab,ti
3396544 records

[Study design filter]
#3 'clinical trial'/exp OR 'controlled clinical trial'/exp OR 'randomized controlled trial'/exp OR 'case control study'/exp OR 'cohort analysis'/exp OR (clinical AND trial) OR trial OR 'controlled clinical trial' OR randomized OR randomised OR rct OR 'case control' OR cohort*
6210143 records

#4 'review'/exp OR 'systematic review'/exp OR 'scoping review'/exp OR 'meta analysis'/exp OR 'case report'/exp OR 'editorial'/exp
7807715 records

[Combination search]

#5 #1 AND #2
16478 records

#6 #3 AND #5
5439 records

#7 #6 NOT #4
4111 records

#8 #7 NOT animal*
3794 records

#9 #8 NOT child*
3267 records

#10 #9 AND [embase]/lim NOT ([embase]/lim AND [medline]/lim)
1501 records

#11 #10 AND ('Article'/it OR 'Article in Press'/it)
287 records

Filters Publication date 2000-2026
276 records

Filter English
254 records

CINAHL Ultimate (EBSCOHost, inception to present)
Date of search: January 28, 2026

[Population/Problem]
#1 MH "Irritable Bowel Syndrome" OR MH "Irritable Bowel Syndrome/DH" OR MH "Brain-Gut Axis" OR XB (Irritable Bowel Syndrome OR IBS OR DGBI OR “disorder of gut-brain interaction” OR Functional Gastrointestinal Disorder* OR FGID OR (Brain-gut AND (interaction OR disorder* OR dysfunct*)))
8732 records

[Intervention/Exposure]
#2 MH "Diet+" OR MH "Eating Behavior+" OR MH "Diet Therapy+" OR MH "Diet, Low Carbohydrate" OR MH "Mediterranean Diet" OR MH "Diet, Gluten-Free" OR MH "Low FODMAP Diet" OR MH "Nutritional Status" OR MH "Malnutrition+" OR XB (Diet* OR eating OR meal* OR nutrition OR nutritient* OR malnutrit* OR food* OR FODMAP* OR low-FODMAP OR (Fermentable AND (saccharide OR oligosaccharide OR disaccharide OR monosaccharide)) OR LFD OR carbohydrate* OR mediterranean OR starch OR sucrose OR ultra-processed OR gluten* OR lactose* OR dairy* OR fiber*)
496641 records

[Study design filter]

#3 (Clinical trial OR trial OR “controlled clinical trial” OR randomized OR randomised OR RCT OR case-control OR “case control” OR cohort*)
1091122 records

#4 “Systematic review” OR review OR “scoping review” OR “meta analysis” OR metaanalysis OR meta-analysis OR editorial OR “case report”
1045253 records

[Combination search]

#5 #1 AND #2
2201 records

#6 #5 AND #3
532 records

#7 #6 NOT #4
364 records

#8 #7 NOT animal*
352 records

#9 #8 NOT child*
308 records

Filter Peer review, Publication date 2000-2026, English
284 records

Total number of records from database searches before deduplication: 1517
