# Supplementary figures and images for: Types and Outcomes of Dietary Interventions in IBS: A Scoping Review
Source: Nutrients. 2026 Apr 23;18(9):1334. doi: 10.3390/nu18091334 (PMC13164966; doi:10.3390/nu18091334)

## Types and outcomes of dietary interventions in IBS: A scoping review

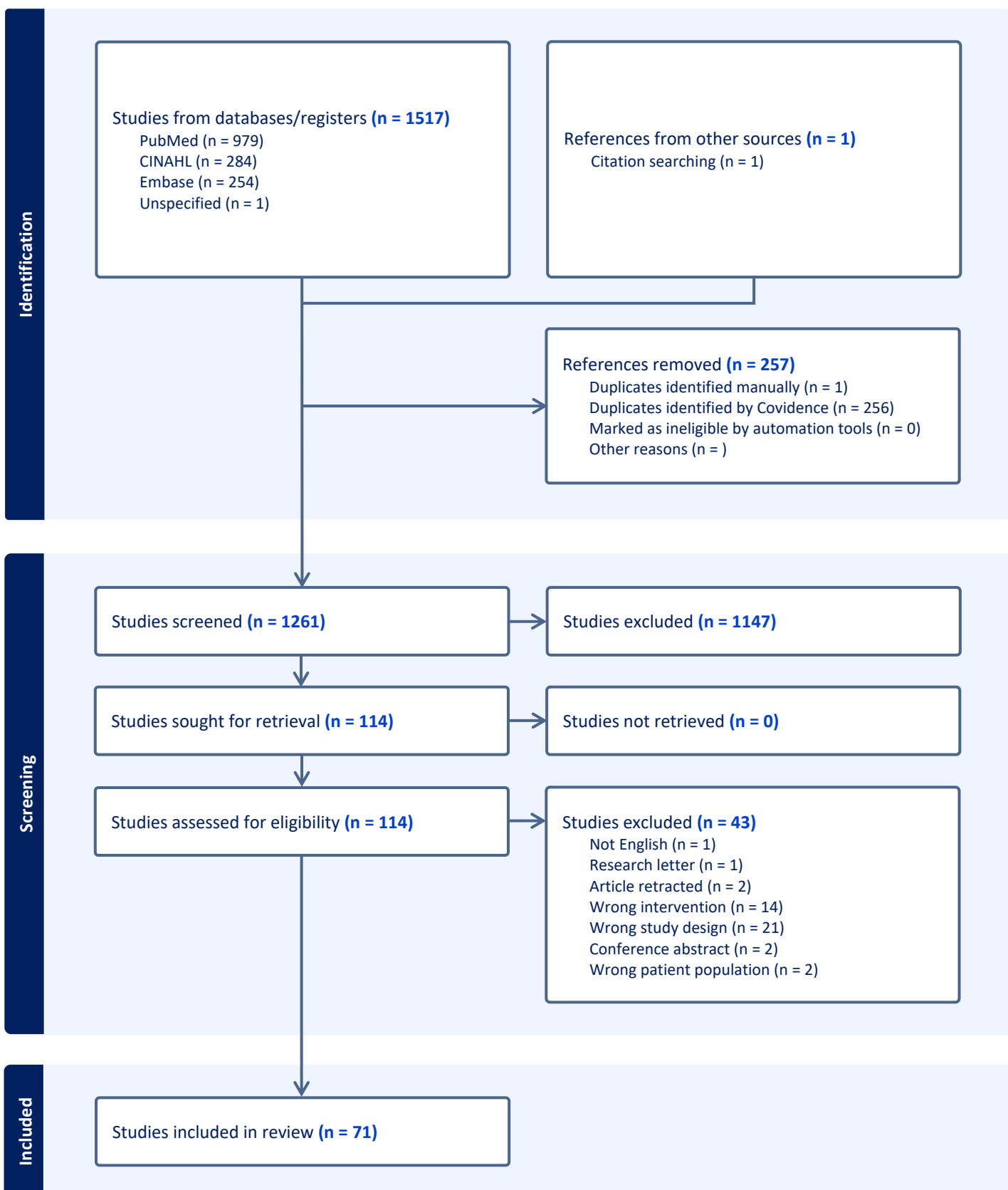

Figure S1. PRISMA flow chart

Supplement: Supplementary file 1 [file nutrients-18-01334-s001.zip › Figure S1 PRISMA flow chart.pdf]
